# Exploring Parental Experiences of Childhood Ear Health Clinics and Their Acceptability of AI‐Based Diagnostic Tools: A Qualitative Study

**DOI:** 10.1111/hex.70421

**Published:** 2025-09-02

**Authors:** Jacqueline H. Stephens, Celine Northcott, Amanda Machell, Trent Lewis, Eng H. Ooi

**Affiliations:** ^1^ Flinders Health and Medical Research Institute, College of Medicine and Public Health Flinders University Adelaide Australia; ^2^ Pregnancy And Newborn Health, South Australian Health and Medical Research Institute Adelaide Australia; ^3^ College of Science and Engineering Flinders University Adelaide Australia; ^4^ Department of Otorhinolaryngology Flinders Medical Centre Adelaide Australia

**Keywords:** artificial intelligence, diagnosis, ear health, parent experience, qualitative research

## Abstract

**Objective:**

Artificial intelligence and machine learning (AI/ML) algorithms will transform the childhood otitis media (OM) diagnostic experience. However, there is limited data on parents' current experiences within clinical settings, limited research exploring AI/ML acceptability among consumers generally, and none regarding consumer perspectives on its use for childhood OM. This study aimed to explore current parental experiences of, as well as their perspectives on the use of AI/ML in, clinical care for OM in children.

**Design:**

We conducted and thematically analysed semi‐structured interviews with parents of children seen for OM within the ENT or audiology departments of an Australian urban teaching hospital.

**Findings:**

Seven themes were identified: (1) Meeting children's needs; (2) Challenges in accessing and waiting for audiology and ENT care; (3) Urban versus rural healthcare experience; (4) Public versus private health system; (5) Strategies for enhancing paediatric audiology services; (6) Perceived benefits of AI/ML in ear disease diagnosis; and (7) Concerns and considerations regarding AI/ML in ear health diagnosis.

**Conclusions:**

Parents have concerns about the use and development of AI/ML tools, but also acknowledge the potential benefits of such tools for healthcare delivery. Currently, the understanding amongst parents of AIAI/ML/ML tools for OM diagnosis was limited, and more education on the use and development of AIAI/ML/ML for OM is warranted.

**Patient or Public Contribution:**

We did not involve patients or the public in the design of this study. However, three authors have lived experience as parents of children who have had recurrent ear infections.

## 1 Introduction

Otitis media (OM) is one of the most common diseases in young children, with almost all children experiencing at least one episode of OM by their third birthday [1]. Commonly termed an ‘ear infection’, OM is the infection and associated inflammation of the middle ear [2] and is the main reason parents/caregivers (‘parents’) seek healthcare for their children [3]. Parents of children with OM have well‐founded concerns for their children's long‐term hearing health [4], as persistent, untreated OM is associated with hearing loss and can have negative impacts on auditory processing, language and speech development, school readiness, social competence, psychosocial well‐being and sleep [5]. As such, early detection is crucial for ensuring timely and appropriate treatment.

Diagnostic modalities for OM and associated hearing loss include (pneumatic) otoscopy and tympanometry [2]. Otoscopic examination generally identifies inflammation, perforation or bulging of the tympanic membrane (‘ear drum’) as the most common form of assessment due to availability and low cost. Tympanometry objectively measures tympanic membrane and middle ear function [2]. These procedures often require parent assistance to comfort their child and help them stay still [6] and require close interaction between parents and clinicians during OM diagnosis. However, while parent–clinician interactions have been explored in some settings [7, 8, 9, 10], including for children with cochlear implants [11], the in‐clinic experiences of parents of children with OM have received little attention.

Audiology testing requires specialised training to use the devices and interpret the results [12], and an accurate diagnosis of OM depends on the technical skills, as well as the clinical experience, of the practitioners in viewing and diagnosing many different ear pathologies [13]. Specialist otolaryngologists (ear, nose and throat; ENT) and audiologists can provide an accurate diagnosis [14], but access to such specialists is limited, and long wait times can impair timely access to quality care, especially in rural and remote areas [15, 16]. As such, the initial diagnosis for OM largely falls on general practitioners (GPs) and other allied health professionals, who have less clinical experience and formal training [12] and who may fail to correctly diagnose OM. For example, the average correct diagnosis of OM by GPs has been shown to be approximately 50% [17, 18, 19], compared to around 74% by otolaryngologists [18, 20]. Thus, systems to support timely and accurate diagnosis of OM are being increasingly explored, with artificial intelligence (AI; the science of creating systems that mimic human intelligence) and machine learning (ML; the science of training computers to learn from data and improve their performance without explicit programming) being increasingly integrated into clinical care.

Artificial intelligence and machine learning (AI/ML) have the capacity to accurately diagnose OM and increase the ability of clinicians to accurately diagnose children with OM. A recent review of 79 studies which used AI/ML‐based technologies to diagnose, treat or manage OM patients demonstrated the potential for AI/ML to accurately diagnose OM [21]. Based on articles for the diagnosis of OM with otoscopy images (*n* = 43 studies), radiology and pathology (*n* = 8 studies) and tympanometry (*n* = 4 studies) results demonstrated AI/ML was as accurate as the clinician or even better, accurately diagnosing OM in up to 99% of cases [21]. While promising from an accuracy standpoint, thus far, there is limited evidence exploring the acceptability of AI/ML among consumers [22], especially regarding its use in the diagnosis of childhood OM.

Given that AI/ML have the potential to transform the OM diagnostic experience for parents and their children, but that there is a paucity of data on what these current experiences entail, we undertook a study to address two interrelated questions. Firstly, to understand parents' current experiences, we sought to answer: *What are parent and caregiver experiences of clinical care of childhood OM*? This then gave context to answer the following research question: *What are parent and caregiver opinions on the use of AI/ML in the clinical care of childhood OM*? Therefore, this study aims to explore consumer perspectives, specifically parental/caregiver perspectives, on the acceptability of using ML for the diagnosis of childhood OM, and examine parental/caregiver experiences during audiology and ENT appointments to contextualise the current landscape of paediatric audiology.

## 2 Methods

We conducted a qualitative study between November 2022 and April 2023 using semi‐structured interviews with parents and caregivers of children with a history of OM to understand their perspectives on using AI/ML in the diagnosis of childhood OM. The Consolidated Criteria for Reporting Qualitative research (COREQ) checklist was used for reporting purposes [23]. Ethical approval was gained from the Southern Adelaide Local Health Network (Project ID: LNR‐56.22ERP) and Flinders University Human Research Ethics (Project ID: 5651) Committees.

### 2.1 Study Setting

Flinders Medical Centre is the main public teaching hospital servicing the southern region of Adelaide. The 560‐bed hospital provides care for around 341,000 people living in the region, including audiology and ENT services for paediatric patients.

### 2.2 Participants and Recruitment

Purposive sampling was utilised to ensure diverse representation among participants, aiming to obtain a wide range of views. Parents and caregivers were eligible for inclusion in this study if they were aged over 18 years; had a child(ren) who presented with OM at the Flinders Medical Centre ENT or Audiology outpatient clinics, and who could read and speak English. Clinic staff introduced the opportunity to participate in the research project with parents and caregivers who met the inclusion criteria and introduced the research officer (C.N.), who provided more information about the study, including providing a hard copy of the parent information and consent form for their review. Potential participants were followed up via email and/or telephone (depending on their preferred method of contact) to ascertain their interest in participation and, following provision of written informed consent, to arrange an interview time and date. Participants were recruited until data saturation was achieved in the qualitative data.

### 2.3 Researcher Reflexivity

This study was conducted by a multidisciplinary team. Two authors (C.N. and A.M.) are public health researchers with expertise in child health and qualitative research methods. A data scientist with expertise in ML (T.L.) contributed technical insights, while clinical expertise was provided by an ENT surgeon and clinician‐researcher (E.H.O.). The first author (J.H.S.), who is an experienced epidemiologist and health services researcher, provided methodological oversight of the study. The authors are parents and caregivers to children who have experienced OM. We acknowledge that our range of professional backgrounds and lived experiences shaped the conduct of this study and our interpretation of the data. Regular team discussions and collaborative analysis were used to challenge our assumptions, and we aimed to centre participant voices while maintaining critical awareness of our positionalities and potential influence on data collection and interpretation.

### 2.4 Data Collection

Semi‐structured interviews were utilised to elicit parent and caregiver perspectives about the acceptability of using AI/ML in the diagnosis of childhood OM. A topic guide (Table 1) was developed based on a review of current literature reporting consumer opinion of the application of ML in general/other healthcare settings [24]. In addition, participants were asked about their relationship to the child (mother, father and other) and their residential postcode. The Index of Relative Socio‐economic Advantage and Disadvantage (IRSAD) produced by the Australian Bureau of Statistics was used as a proxy measure of each participant's socio‐economic status [25].

**Table 1:** Topic guide developed for the semi‐structured interviews.

Topic	Guide for discussion
Paediatric audiology services	General experience Ideas for improvement
ML and AI as a general concept	General understanding of artificial intelligence and machine learning
ML for the detection of childhood ear disease	Thoughts on how AI/ML could be used for the detection of childhood ear disease Benefits and concerns of AI/ML for childhood ear and hearing health screening procedures

For consistency, interviews were conducted by one researcher (C.N.) at a time and date convenient to the participant, either face to face or by telephone. Interviews began with verbal reaffirmation of consent, followed by a general discussion of parent experiences of paediatric audiology services and suggestions for improvement, before moving into a discussion of parent views on AI/ML. During the discussion of AI/ML for ear health, some examples of potential AI/ML applications were provided, such as the use of otoscopic images in AI/ML algorithms. Following each interview, the researchers (C.N. and J.H.S.) met to debrief about the topics raised by the interviewee and to review data saturation. Using the constant comparative method [26], after each interview, we reviewed the predictability and consistency of interviewee responses to determine when new interviews reinforced existing themes, rather than introduced new ones [27]. Rather than only seeking code saturation, we also sought ‘meaning saturation’ [28], which was deemed reached when additional interviews stopped producing new insights or understandings.

Interviews lasted between 9 and 25 min (mean duration = 19 min), were audio‐recorded, and subsequently transcribed. We invited parents to review their interview transcripts to ensure correct meanings are captured; however, none of the parents chose to do so. Small thank‐you gifts, in the form of a $25 gift voucher, were provided to participants to thank them for their time and for sharing their knowledge.

### 2.5 Data Analyses

Guided by grounded theory, an iterative analysis of the transcripts was used to synthesise data into themes [29, 30, 31]. Grounded theory operates within a constructivist paradigm, rejects ideas of emergence and objectivity of analysis, and instead embraces the researcher's subjectivity in findings. Therefore, the researcher's reflexivity was a critical component of the analysis process. Rigour was increased by multiple researchers undertaking analysis [32]. Three researchers independently coded the transcripts (C.N., A.M. and J.H.S.). Data analysis began with a familiarisation stage, wherein the research team discussed the common experiences shared by parents. NVivo software (QSR International Pty Ltd. Version 1.7.1) was used to organise data with a constant comparative approach, followed throughout the stages of initial coding, focused coding and theoretical coding procedures. Initial coding was done line‐by‐line, remaining close to the data, and maintaining participant wording in code generation where possible. Following code identification, code relationships were synthesised through consensus discussions and mind mapping into themes and sub‐themes. Final themes and sub‐themes, as well as their relationality, were agreed upon following the roundtable consensus.

## 3 Findings

Of 26 parents and caregivers who were invited and indicated an interest in participating, 10 consented and participated (38.5% response rate). As described in the methods, participants were consented and interviewed until data saturation was achieved (*n* = 10). All participants were parents of the child seen in the audiology or ENT clinic, with nine identifying as the child's mother and one as the child's father. Five participants resided in areas considered socio‐economically advantaged (IRSAD decile 8) and four participants lived in moderately advantaged areas (IRSAD 6 or 7), while one participant lived in an area considered amongst the most disadvantaged (IRSAD decile 2).

**Figure 1:**
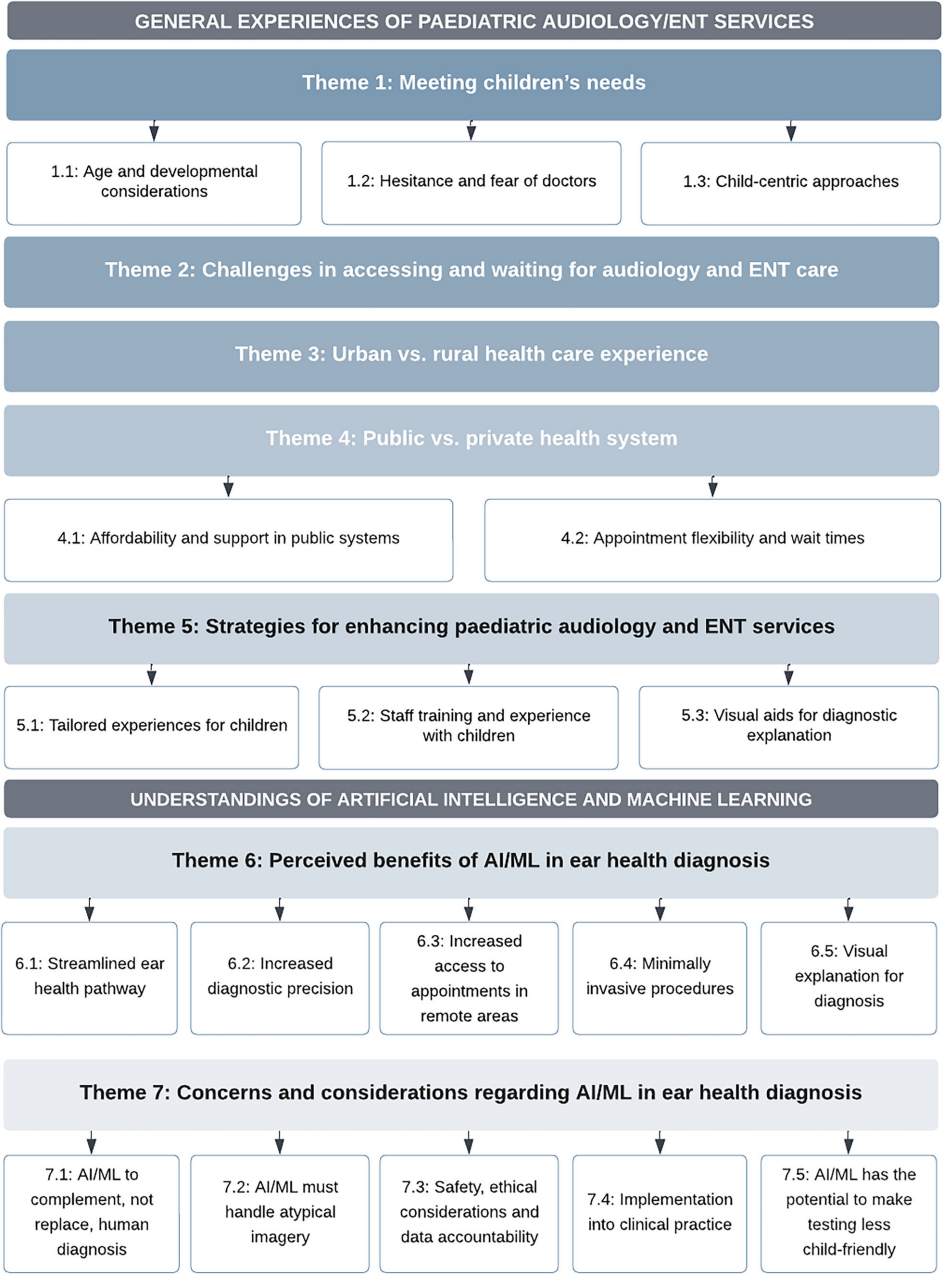
Schematic representation of key concepts identified in the parent interviews.

The interviews generated qualitative data which could be distinctly separated into two components: (1) general experiences of paediatric audiology and ENT services and (2) understandings and perceptions of AI/ML for ear and hearing healthcare. These two components, and the themes and sub‐themes identified for each (Figure 1), are discussed in detail below.

### 3.1 General Experiences of Paediatric Audiology and ENT Services

Several themes were identified when examining the experiences of parents navigating audiology and ENT appointments with their children. The first identified theme encompassed the necessity to accommodate children's needs, particularly the needs specific to the child's age and developmental stage, as well as hesitancy and fear of doctors, and ensuring child‐friendly approaches. The second theme reflected the frustrations voiced by parents on prolonged waiting times and the inherent distress surrounding the uncertainty of diagnoses. The third theme focused on geographical disparities between audiology care in rural versus urban settings, with a fourth theme focused on the disparities between public versus private audiology and ENT services. The final theme was strategies suggested by parents for enhancing paediatric audiology services.

#### 3.1.1 Theme 1: Meeting Children's Needs

Throughout the interviews, parents consistently highlighted the importance of addressing their children's specific needs when presenting to audiology care, delineating three sub‐themes: age and development, hesitance and fear of doctors, and child‐centric approaches.

##### 3.1.1.1 Sub‐Theme 1.1: Age and Developmental Consideration

When discussing their experiences of paediatric audiology services, four parents spoke of age and development‐related challenges. Appointments with younger children were considered more challenging than appointments with older children, and quick appointments were considered good for young children due to short attention spans. The following quotes illustrate this:

It might be because he is older, but they'd put the earphones on and then try and do the graph thing to test, but when he was two, he didn't really like that. But when he was four, he did and participated…

(P1)

…It was really quick, which was good, because the attention span of a two‐and‐a‐half‐year‐old isn't so great.

(P2)

The parent of one child with learning and developmental delays spoke of the challenges in obtaining an accurate diagnosis. They highlighted a discrepancy between the expectations of the hearing test and their child's developmental capabilities, noting a gap between what the audiologist anticipated and what their child could feasibly accomplish, as follows:

I just didn't feel like it necessarily worked that well. I just didn't think that ‐ like there was just a gap between what they were expecting of him and what he actually had capacity to be able to do.

(P8)

##### 3.1.1.2 Sub‐Theme 1.2: Hesitance and Fear of Doctors

Three parents recounted instances where their children exhibited hesitance to having their ears examined and described the need to hold their children down so the audiologist could perform the assessment. One parent described their child's apprehension as upsetting:

…she initially was very, very hesitant, we had to practically hold her down…. Yeah, it really upset her the first few times…

(P7)

Additionally, another parent spoke of their child's fear of the doctor's appearance, particularly noting discomfort with the use of magnifying goggles:

And he wears like those goggle‐y glass things, you know, with I don't know what, magnified, kind of around his neck. And I must admit [child] was a bit more hesitant. He thought that looked like something scary and didn't really like it…

(P5)

##### 3.1.1.3 Sub‐Theme 1.3: Child‐Centric Approaches

Expanding on the theme of catering to children's needs, five parents described their experience as more positive when audiologists tailored their appointments to suit their child's preference. By engaging in child‐centric practices such as asking ‘…*questions about what his interests were at the time…*’ (P2) and using ‘*…coloured, flavoured tongue depressors…*’ (Int 5), audiologists fostered a more welcoming and engaging environment. Parents expressed appreciation for these personalised approaches, noting a positive experience for their children. One parent remarked, ‘*[audiologist] was really good. It really, also does depend on the audiologist and if they can grasp kids and are good at dealing with children as well*’ (P1). Another parent echoed this sentiment, stating: ‘*[audiologist] is so kind and was patient with kids*’ (P3).

#### 3.1.2 Theme 2: Challenges in Accessing and Waiting for Audiology and ENT Services

During the interviews, another prominent theme that emerged was the sense of frustration experienced by parents navigating and accessing audiology care, particularly concerning extended wait times and delays in receiving diagnoses for their children. Notably, two parents spoke about the frustration associated with prolonged waits in the clinic. One parent particularly expressed frustration with receiving a ‘wait and see’ diagnosis. This parent voiced concern about the potential consequences of delayed treatment for their child, further compounded by the logistical challenges imposed by needing to take time off work to attend appointments. The following statement illustrates this frustration:

‘…He [doctor] was just like we'll just wait and see. And just after spending over an hour waiting just for him to literally just look at him for two seconds and go, “We'll see him again in a few months”, it was just really frustrating because I'm just like, well, how many times is his eardrum going to burst again in those times? It's just pushing treatment back further and further … and I guess we were worried about the permanent damage it could possibly do to him … and I guess my worry was not only … having these infections, which I was assuming he was going to do; are we just going to get the same answer in another 3 weeks, are we going to spend our time—potentially my partner needs to take more time off work—coming in and then just be like, wait and see again.

(P9)

#### 3.1.3 Theme 3: Urban Versus Rural Healthcare Experience

Parents voiced concerns about the noticeable differences between audiology care provided in urban and rural areas. One parent noted a preference for driving to the city for appointments due to negative experiences in their country town and a perception that clinic staff are better trained in the city. The parent gave an example of receiving conflicting results for the same test, with a perception that there were differing outcomes between testing in the city and the country, which raised doubts for the parent about the accuracy of diagnoses between the two locations. However, the authors acknowledge OM evolves and changes over time, so it is possible there was no effusion when first tested, but one developed later and was present for later testing. The parent's quote is as follows:

…It [test done in country town] was meant to be the exact same hearing test that he had in Adelaide [city] … [but] … it came back that his hearing was fine. But … we booked him in to have the grommets anyway, and … when [doctor] put the grommets in, he said, “He definitely had a build‐up of glue in the ear and it definitely needed to be done". And if we had of gone with the [result of the] second hearing test [in the country town], we wouldn't have done it … this is why my husband and I always go to Adelaide and Melbourne for health reasons, because we've had such bad experiences in our country town, … we go and seek this advice from specialists and these clinics in the cities, because I feel like their staff are better trained and have better equipment … I don't believe they have as much training or they don't have as much of an opportunity for courses or attending courses, because we are quite isolated.

(P6)

#### 3.1.4 Theme 4: Public Versus Private Health System

Parents frequently drew comparisons between the public and private health systems, highlighting the affordability and perceived effectiveness of the public system, although at the expense of long wait times and a lack of choice over appointment times.

##### 3.1.4.1 Sub‐Theme 4.1: Affordability and Support in Public Systems

Parents reflected on their experience in both the public and private systems, explaining that the private system is like a business and the public system is cheaper and more helpful. One parent stated, ‘… *the next time, what we've done [public] amazing and cheaper*’ (P1). Another parent stated:

I've been like public … and I've also been [private]…. And they are both extremely different, how they operate. And one, I feel like, overall, is more helpful and just wants a good outcome. The other one, I feel like, now that I know the other end of it is like a business and they just want your money…

(P5)

In contrast, others preferred to pay private appointment fees, noting ‘… *we saved our money to see [doctor in private system] so it wasn't an easy option, but it was something we could do. But some people literally wouldn't have the $160 or however much it is …*’ (P9).

##### 3.1.4.2 Sub‐Theme 4.2: Appointment Flexibility and Wait Times

Parents also expressed frustration with the limited options for appointment scheduling and extended wait times in the public health system. One parent stated: ‘*but once we were in the public system, it's just that waiting game. And you don't have as much choice over appointments, which we've got four kids so it just sometimes gets a bit hard because they've all got appointments so I know some days [husband] has just had to take half a day off work just so I could get him to an appointment because the other kids have got other stuff to do. So, I think that's just probably the harder bit around the public system; that less choice*’ (P9).

#### 3.1.5 Theme 5: Strategies for Enhancing Paediatric Audiology and ENT Services

During interviews, parents shared their thoughts on how audiology and ENT services could be improved for children. Three key improvement strategies were identified by parents: (1) the importance of tailoring audiology care to children, (2) enhancing staff training to better engage with children and (3) the integration of visual aids to facilitate better understanding of their child's diagnosis. As expected, these suggested improvement strategies closely align with their experiences, described above.

##### 3.1.5.1 Sub‐Theme 5.1: Tailored Experiences for Children

During the interviews, parents consistently highlighted the need to tailor the audiology experience to meet the needs of the child. Parents expressed the desire for appointments to be more child‐friendly, noting the experience ‘*should have been easier*’ and ‘*funner [sic]*’, and that the rooms, gowns and doctors' appearance should be ‘*colourful, bright, kid‐like’*.

##### 3.1.5.2 Sub‐Theme 5.2: Staff Training and Experience With Children

The second strategy was centred on staff training in paediatric audiology care and experience with children. Suggestions for improvement included that the clinician needed ‘*to be someone with experience [with children]*’ and that ‘*more training for staff in country locations’* was needed. Also, continuity of care was identified as missing for children with recurrent ear disease, with one parent describing the experience as being referred ‘*to lots of different doctors and I got fobbed off quite a bit’.* Parents stated that better training for GPs was needed to improve the diagnosis and referral process in primary care settings.

##### 3.1.5.3 Sub‐Theme 5.3: Visual Aids for Diagnostic Explanation

The third strategy focused on the need to provide visual aids and more extensive explanations during appointments to help parents and children understand ear disease and the reasons for treatment options. One parent stated:

For the diagnosis, with the ear stuff, because it's the doctor looking inside the ear canal, it's not like when you get an x‐ray and you can point to, well here's X and you can see X doing this and this and therefore you have to remove it or you have to do this to it to make it work. It's from my perspective, a little bit more subjective, and I'm a little bit more relying on the doctor's word rather than what I can see for myself.

(P4)

Further highlighting this, another parent noted: ‘it would've been good if he explained why he wanted to wait another few months to wait and see, because I'd seen him [the child] in pain multiple times and it wasn't fixing itself so I couldn't understand why he thought it was a good idea just to wait and see.

(P9)

### 3.2 Understandings of AI/ML

Parents were asked to share their general perceptions of AI/ML and then think about these concepts in relation to healthcare settings. Parents associated AI/ML with robots and machines. Other responses included ‘…something that's not real’ (P2), ‘…not something that we know for sure to be true’ (P8) and ‘…it's not an intelligence as such’ (P4). None of the parents had heard of AI/ML being used in ear health diagnosis, but some had heard of AI/ML in other areas, including skin cancer diagnosis, literacy and numeracy assessments, and apps for diagnosing plant disease. In addition to these general perceptions of AI/ML, parents expressed opinions on potential benefits and concerns of using AI/ML in ear and hearing healthcare. As a result, two themes were identified: (1) Perceived benefits of AI/ML in ear disease diagnosis and (2) Concerns and considerations regarding AI/ML in ear health diagnosis. These themes, and their sub‐themes, are presented in detail below.

#### 3.2.1 Theme 6: Perceived Benefits of AI/ML in Ear Health Diagnosis

Parents identified five potential benefits of using ML for childhood ear health diagnosis. These were: (1) the potential to streamline the ear health pathway, (2) increased diagnostic precision, (3) increased access to appointments for people living in remote areas, (4) ability to shift to minimally invasive procedures; and (5) the potential of AI/ML to provide a visual explanation of the diagnosis.

##### 3.2.1.1 Sub‐Theme 6.1: Streamlined Ear Health Pathway

Parents suggested that AI/ML for ear health diagnosis would increase efficiency through the ear health referral pathway. They stated that AI/ML would benefit from supporting early diagnosis and intervention. As illustrated in the following quotes:

So, she wouldn't have to go multiple times? If they could tell straight away with one image and compare it, yes, she needs grommets again. Instead of, yes, she needs grommets and then you've got to go here, here, here, and here, and then we'll give you the grommets.

(P7)

And I think that it would ultimately help diagnose children potentially earlier, which is the key. If you can nip it in the bud before hearing loss occurs or before a child gets these recurrent ear infections or is snoring or is having sleep apnoea, all of those different things … the early intervention…

(P10)

##### 3.2.1.2 Sub‐Theme 6.2: Increased Diagnostic Precision

Parents viewed diagnoses using AI/ML as being more accurate than diagnoses by human diagnosticians (audiologists and ENT surgeons). AI/ML diagnosis was seen as a potential way to compensate for insufficiently trained healthcare workers, as well as an option for the diagnosis of children with language or developmental delays who may not have the ability to follow complex audiology test instructions. For example, one parent (P8) stated: ‘*…I guess it's probably more accurate than relying on [person]’*. Another parent with experience in data management stated:

The data person in me says, “I reckon they get it right more than they get it wrong.” A surgeon's probably going to get it right 80% of the time. I think a machine that's been trained properly will probably get it right, 90%.

(P4)

The following quote from a parent illustrated the perception that the use of AI/ML in ear disease diagnosis could lead to more accurate and easier hearing testing for children who do not have the ability to follow the testing instructions. However, the parent discusses concepts which may be outside the current AI/ML scope for ear testing:

…It's just that he wasn't able to verbalise or acknowledge personally himself through his own actions that he was hearing [the audiology test]. So, if there's some ways of recognising, I don't know, whether it's his heart rate or whether it's something that kind of you can acknowledge is changing in him, that would help to sort of get a more accurate read, I guess.

(P8)

##### 3.2.1.3 Sub‐Theme 6.3: Increased Access to Appointments in Remote Areas

Parents spoke about how ML could increase access to appointments for people living in remote areas. As stated by one parent (P1): ‘*I actually think that would be great and especially for people in remote areas that can't access normal appointments and stuff like tha*t.’ Another parent discussed how AI/ML could facilitate decision‐making between local healthcare providers in primary care settings, such as a GP, with a more specialised clinician, such as an ENT surgeon. The following quote illustrates this:

I can see how it could work, though if you lived a thousand kilometres away from the specialist … when you've got a local country GP, when they could get some type of inner ear photo to send, I can definitely see the benefit of that.

(P7)

##### 3.2.1.4 Sub‐Theme 6.4: Minimally Invasive Procedures

AI/ML was considered by parents to be a non‐invasive way to perform ear health diagnosis and stated this was a more child‐friendly option and preferable for children compared to current testing options, as stated in the following:

I think it'd be great. It's less invasive really. It's a photo. As opposed to what they go through, sticking things in ears, listening to things. Especially if kids don't like hearing earphones on and stuff like that.

(P1)

##### 3.2.1.5 Sub‐Theme 6.5: Visual Explanation for Diagnosis

The potential of AI/ML in ear health diagnosis to be used to support the clinical care discussion was identified. The ability for AI/ML to be used to offer visual aids, such as allowing the parent to see the tympanic membrane, would assist clinicians in their explanations and reasoning to parents for a particular diagnosis. The ability to more fully include parents in these clinical discussions was encouraged by parents, and they expressed that they wanted to be more informed about their children's diagnosis and care. One parent explained this as follows:

If it's something that they could pull up on the screen and actually show us, it might be a point of conversation where that particular time when he didn't explain why, maybe it's because he had a look at it and obviously it's not something that I can see so he didn't think to discuss it. Whereas if it was something he could show me, he could have said potentially, you know, this is why we're putting it off or just being able to see what they see and being able to explain it a bit better.

(P9)

#### 3.2.2 Theme 7: Concerns and Considerations Regarding AI/ML in Ear Health Diagnosis

Parents expressed several concerns about the potential use of AI/ML for childhood ear health diagnosis, parents cited concerns about AI/ML replacing, rather than complementing human diagnosis; questioned the ability of ML to handle atypical diagnoses; were concerned about the safety and privacy of ear health data; questioned how it would be implemented and by whom; and had concerns about the potential for AI/ML tools to make testing less child‐friendly.

##### 3.2.2.1 Sub‐Theme 7.1: AI/ML Must Be Complementary to, Not Replace, Human Decision‐Making

Parents stated AI/ML should complement, rather than replace, human decision‐making about ear diagnoses. Parents expressed the importance of maintaining human interaction in the diagnostic process and had apprehensions about the potential for diagnosis to be based solely on imagery, raising concerns about loss of human oversight. One parent asked:

Is the intention that there's no human interaction at all? … I feel like a parent has to go to a doctor and say, my child is experiencing a problem with X. And then that starts the diagnostic process, right? I can't see myself going to a machine and saying, my child is experiencing a problem with X. Like there still needs to be some sort of gatekeeper, I guess.

(P8)

Another parent raised concern about the potential for AI/ML to ‘lead to an incorrect diagnosis’ (P10) and discussed the need for AI/ML to be used as part of a suite of testing tools.

##### 3.2.2.2 Sub‐Theme 7.2: AI/ML Must Be Ability to Handle Atypical Imagery

Parents expressed concern regarding the ability of AI/ML to handle atypical imagery. For some parents, this concern stemmed from an apparent gap in understanding ear anatomy and disease progression, as well as how AI/ML algorithms are developed. While the following quote is from a parent questioning the ability of AI/ML to successfully image the tympanic membrane during an ear infection, the quote also illustrates a knowledge gap about OM:

It is how clear you can get the picture as well, whereas she has had so much fluid and how clearer the picture are you able to get?

(P7)

##### 3.2.2.3 Sub‐Theme 7.3: Safety, Ethical Considerations and Data Accountability

Parents raised concerns regarding the safety and ethics of implementing AI/ML tools in ear disease diagnosis. Specific safety concerns were often not articulated by parents, instead being discussed as a general concern, for example: ‘*I think about if he would be safe or not*’ (P3). When parents did specify their safety concern, it was the privacy of their child's data. These privacy concerns were focused on consenting processes for using imagery in an AI/ML tool, either for the diagnosis of disease in their own child or to be incorporated into a dataset for testing and training during the AI/ML development process. As stated by one parent when discussing how they felt about their child's data being used to develop AI/ML tools: ‘*If it's just a photo of the ear and it won't be identified*’ (P4). Another parent reflected on their own position on their child's data being used in AI/ML applications, as well as how they perceive other parents may feel:

…if someone asked me if we can use this information, I'm happy to use it, but I understand why others wouldn't be. I'm sort of blindly working on the assumption that the data is collected ethically and that people that don't want their information used in that way are free to make that choice for themselves.

(P8)

Parents emphasised the importance of accountability in how AI/ML is used and by whom. They also discussed where the accountability for the child's ear disease diagnosis would be placed, ultimately stating the responsibility for diagnoses should lie with the practitioner diagnosing the child. As stated by a parent:

I would think that it [accountability] would still be the practitioner or the person that is the professor, the doctor, whoever it is that's making the diagnosis; that would be on them…

(P10)

##### 3.2.2.4 Sub‐Theme 7.4: Implementation into Clinical Practice

Parents questioned the way AI/ML would be implemented into clinical practice. Parents stated the person using the AI/ML tools needed minimum qualifications, with parents noting the importance of a medical background. An assumption that specialists would use AI/ML tools was noted, further reinforcing the rhetoric on the importance of medical qualifications. The following quotes illustrate this perception:

It wouldn't be just some random trainee using a machine. It would have to be someone … with some … medical background…

(P4)

I know these machines would be used by specialists and overseen by specialists in the cities.

(P6)

When considering the implementation of AI/ML in regional locations, parents had the opinion that the interpretation of results needed to be checked or confirmed by a specialist, with one parent positioning specialists as being in the city, as stated in the above quote: ‘… overseen by specialists in the cities’. Parents also discussed that the implementation of AI/ML for ear health screening should be part of routine practice, such as routine child health and development checks or as part of the immunisation schedule. Once, a parent stated:

…this could be a part of the early childhood—you know how you've got your 3 months, 6 months, 9 months check‐up. It could be done in collaboration with immunisations for example.

(P10)

##### 3.2.2.5 Sub‐Theme 7.5: AI/ML Have the Potential to Make Testing Less Child‐Friendly

Rather than improving the patient experience, some parents raised concerns about the potential for AI/ML tools to increase the volume of testing children would be required to undergo. One parent queried ‘*how often would [children] have to have these pictures?*’ and went on to say, ‘*…her ear infection this week might be a completely different type of ear infection she has in a few weeks' time … would she need two different photos; do you know what I mean?… Is it really replacing a doctor looking in there?*’ (P7). In addition to this, parents were concerned that ear health tests using AI/ML tools may add to the anxiety children already have about undergoing testing. One parent raised the concern that an AI/ML ‘machine’ had the potential to frighten children.

For some kids, they might be scared if it's just another bit of machine coming at them.

(P9)

## 4 Discussion

AI/ML are rapidly advancing tools with demonstrated significant potential for identifying OM, as well as enhancing clinicians' diagnostic capabilities for children with ear and hearing conditions [21]. Herein, we present qualitative insights from parents and caregivers of children affected by OM, exploring both their current experiences of accessing audiology and ENT care and their perspectives on the introduction of AI/ML into these settings. Parents described familiar challenges in navigating care—including wait times, fragmented communication and variability in access—reflecting previously reported literature [4, 33]. Within this context, parents reflected on how AI/ML may reshape clinic experiences, their perceptions of the acceptability of AI/ML integration into ear and hearing health diagnostics, along with considerations for its implementation and scalability into routine clinical practice, including suggested strategies for clinical care improvement.

### 4.1 Current Experiences in the Clinic

Meeting the specific needs of children in audiology and ENT appointments is a priority; clinicians need to tailor their approaches based on the child's age, developmental stage and emotional responses. Notably, younger children require shorter, more engaging appointments due to their limited attention spans compared to older children, who can typically navigate more complex testing procedures. The discrepancy between the audiologist's expectations of children and the developmental capabilities of children who may exhibit developmental delays also highlights the need for more adaptable and developmentally appropriate diagnostic methods. Likewise, parents' descriptions of the hesitance of young children to engage with diagnostic audiology tests, perhaps due to fear of medical equipment or unfamiliar environments, suggest child‐friendly spaces, tools and personalised care could improve cooperation during appointments. This builds on prior research which supports family‐centred care [34, 35, 36], that is, care which ensures a family's needs, preferences, cultural background and values are respected and integrated into the care process [33, 37, 38], and whereby tailoring medical interactions to the child's developmental stage and emotional needs can enhance both the child and the family's experience [35], as well as lead to better health outcomes [36].

Adding to the barriers of audiology care, prolonged wait times and delays in diagnosis are also major concerns [4], not only causing emotional distress for families but may lead to poorer behavioural outcomes [39]. While delayed antibiotic treatment is typically part of routine practice in managing OM to prevent antibiotic resistance, timely evaluations are still important to ensure appropriate management and avoid complications [40]. Disparities in audiology and ENT care between urban and rural areas were another concern, with parents expressing a lack of confidence in healthcare offered in rural settings. The perception that there is better training and equipment in urban areas highlights a clear need to improve the provision of care in rural areas, potentially through increased staff training and investment in better diagnostic tools. This sentiment was echoed in the public versus private health system in Australia, with parents noting distinct advantages and challenges associated with each. While the public system is appreciated for its affordability, the long wait times and limited appointment flexibility remain significant drawbacks for parents. On the other hand, private healthcare, despite offering shorter wait times and greater flexibility, is expensive and often inaccessible for many families. These issues are particularly critical given the importance of timely management of OM [39, 41]. However, paediatric ear health services are often not readily available due to the limited capacity of specialists and the high demand for audiology and ENT appointments [42, 43].

Taken together, the experiences of parents and caregivers of children receiving audiology care emphasise the need to overhaul the way ear healthcare pathways and diagnostics are provided. The use of AI/ML offers a promising solution. Despite a significant gap in parents' understanding of AI/ML, they generally accept its use in diagnosing childhood OM when the concept is explained to them. Parents perceive AI/ML to offer several benefits, including enhanced diagnostic accuracy and clearer explanations through visual aids. Moreover, it has the potential to streamline the diagnostic process, leading to timely interventions and fewer appointments. Integrating this technology could also reduce wait times and increase access to timely and appropriate diagnoses, particularly those in rural areas with limited access to specialists.

### 4.2 Reshaping the Clinic Experience With AI/ML

Given these insights, the integration of AI/ML in ear health diagnostics appears not only feasible and well‐accepted but also highly beneficial for both patients and the healthcare system. However, successful implementation requires addressing several concerns and complexities associated with AI/ML technology within the paediatric population.

One key aspect is ensuring that AI/ML complements rather than replaces human diagnosis, which is crucial not only for maintaining the safety and emotional comfort of children but also for preserving diagnostic accuracy and human interaction in healthcare. Research by Sezgin et al. (2023) [44] supports this, indicating that AI/ML should enhance diagnostic accuracy as a supportive tool rather than substituting clinician judgement. This is particularly true when handling atypical cases. Studies, such as those by Hallowell et al. (2022) [45], have highlighted the challenges AI/ML algorithms face in detecting rare diseases, highlighting the ongoing need for human oversight.

There are also some concerns about the use and storage of AI‐generated findings, their interpretation, accountability for potential misdiagnosis, and the competency of healthcare professionals using these technologies. These issues are well‐documented in the broader medical field [46], and this study highlights their relevance specifically in paediatric ear health diagnostics. Ensuring that healthcare providers receive adequate training to effectively integrate AI/ML tools into ear health is therefore essential.

### 4.3 Strengths and Limitations

The rigour and validity of qualitative research methods are often questioned, as are the often small sample sizes of the study population. To address this, we endeavoured to undertake data collection until data saturation was reached, and in this study, data saturation was reached very quickly. A study population of 10 parents generated enough data to understand the codes and themes, with no new data generated by additional interviews. To ensure rigour and validity of the research findings, we acknowledged our positionality, reflexivity and potential biases during the research process [47]. We undertook a methodical, predefined research process, including independent iterative coding of the qualitative data to identify the codes and themes, with consensus reached through discussion. Our methods ensure the credibility, dependability, confirmability and transferability of the findings [48]. As such, our findings are confidently reported as representative of the experiences and understandings of parents about audiology and ENT care, as well as the potential use of AI/ML in ear and hearing health diagnostics.

Potential limitations of this study include the low response rate and the self‐selection of participants. Firstly, only 38.5% of participants who indicated an interest in participating ultimately consented to participate in an interview. While face‐to‐face recruitment in clinical trials can have high response rates (> 95%) [49], the consent rate is typically about 68%–72% [50, 51]. In comparison, surveys have much lower response rates, with telephone surveys reportedly having anywhere from 30.2% to 44.1% response rates [52, 53]. As such, our response rate for our telephone interviews, while moderate, is in keeping with the literature reporting response rates for data collected via telephone. Secondly, our participants all spoke English as their main language, were primarily the child's mother, and most resided in areas of moderate to high socio‐economic advantage. As such, the study population is homogeneous, and their perspectives may not reflect those of the general population. Additional research exploring the perspectives of a more diverse participant population is warranted.

### 4.4 Implications for Policy and Practice

Integrating AI/ML into ear health diagnostics presents numerous advantages for parents/caregivers, children and the Australian healthcare system. By leveraging AI/ML technologies, the diagnostic accuracy of ear health could be significantly improved, enabling clinicians to provide timely diagnoses and potentially reducing the need for multiple appointments if early diagnosis and appropriate intervention of OM is provided to children. This proactive approach, particularly beneficial in rural areas, could lessen the travel burden on families and associated financial strain by making diagnostic tools accessible in regions with limited access to ENT specialists. Furthermore, AI/ML tools have the potential to overcome not only geographical barriers but also cultural and language barriers. This could especially be valuable for community workers who may be better suited to providing audiology care than ENT specialists in certain cases. However, the successful implementation and scalability of such initiatives need to consider the ethical implications, proper training of personnel and regulatory frameworks surrounding the use and storage of data for ML, as well as the accountability and decision‐making for medical diagnoses. These issues are important to ensure the responsible and effective integration of AI/ML in advancing audiology care.

### 4.5 Conclusion

Parents and caregivers navigating ear and hearing healthcare for their children often face long wait times, multiple appointments and uncertainty around diagnosis and treatment. Integrating AI/ML tools into the care pathway offers the potential to streamline processes, reduce delays and provide families with earlier clarity about their child's condition. Despite these numerous advantages of AI/ML, integrating such tools into ear health pathways requires strategic policy development to support effective implementation. Policy considerations must address the design, delivery and dissemination of these tools to ensure a responsible rollout. Importantly, parents emphasised that implementation must ensure that new technologies enhance—not replace—the human connection and trust that are central to paediatric healthcare. The scalability of diagnostic tools requires sustainable funding (potentially through public–private partnerships), top‐down advocacy, and education and training for health providers, alongside ongoing collaboration and support from end users, including parents, caregivers and children. Additionally, these AI/ML tools must be relevant and acceptable within the cultural and community contexts where they are implemented. Robust ethical and regulatory frameworks are also essential to safeguard patient privacy, ensure transparency and establish accountability in AI‐driven diagnostic decisions. Clear delineation of responsibilities between diagnosticians and technologists is fundamental for the accuracy and safety of ear health diagnoses moving forward. This study highlights the need for future pilot programs to evaluate the efficacy and real‐world effectiveness of such tools before widespread implementation.

## Author Contributions

**Jacqueline Stephens:** conceptualization (lead), funding acquisition, methodology (lead), formal analysis, project administration, validation, resources, supervision, writing – original draft (lead), writing – review and editing (lead). **Celine Northcott:** investigation, formal analysis, validation, visualization, writing – original draft preparation (lead), writing – review and editing. **Amanda Machell:** formal analysis, writing – original draft, writing – review and editing. **Eng Ooi:** conceptualization, funding acquisition, resources, writing – review and editing. **Trent Lewis:** conceptualization, funding acquisition, writing – review and editing.

## Ethics Statement

Ethical approval was gained from the Southern Adelaide Local Health Network (Project ID: LNR‐56.22ERP) and Flinders University Human Research Ethics (Project ID: 5651) Committees.

## Conflicts of Interest

The authors declare no conflicts of interest.

## Data Availability

The data that support the findings of this study are not publicly available due to privacy or ethical restrictions.
